# Nomogram for predicting urinary retention after radical hysterectomy for cervical cancer with comparative analysis of surgical methods

**DOI:** 10.12669/pjms.41.12.12969

**Published:** 2025-12

**Authors:** Tan Chen, Lan Zhen, Yanzhao Su, Huan Yi

**Affiliations:** 1 Tan Chen, Department of Gynecology Oncology, Fujian Maternity, Child Health Hospital College of Clinical Medicine for Obstetrics & Gynecology, Pediatrics, Fujian Medical University, Fuzhou, Fujian Province 350001, P. R. China; 2 Lan Zhen, Department of Gynecology Oncology, Fujian Maternity, Child Health Hospital College of Clinical Medicine for Obstetrics & Gynecology, Pediatrics, Fujian Medical University, Fuzhou, Fujian Province 350001, P. R. China; 3 Yanzhao Su, Department of Gynecology Oncology, Fujian Maternity, Child Health Hospital College of Clinical Medicine for Obstetrics & Gynecology, Pediatrics, Fujian Medical University, Fuzhou, Fujian Province 350001, P. R. China; 4 Huan Yi, Department of Gynecology Oncology, Fujian Maternity, Child Health Hospital College of Clinical Medicine for Obstetrics & Gynecology, Pediatrics, Fujian Medical University, Fuzhou, Fujian Province 350001, P. R. China

**Keywords:** Cervical cancer, Nomogram, Postoperative complications, Urinary retention

## Abstract

**Objective:**

To identify risk factors for urinary retention after radical hysterectomy for cervical cancer (CC) and establish a nomogram model to facilitate individual treatment decisions.

**Methodology:**

In this retrospective study, records of 438 patients with CC undergoing radical hysterectomy between December 2011 and December 2018 at Fujian Maternity and Child Health Hospital, China were analyzed. Patients were divided into the abdominal operation group (196 cases) and the laparoscopic group (242 cases) based on the surgical method. Short-term outcomes were compared. Independent risk factors for postoperative urinary retention identified by multivariate logistic regression were used to develop a nomogram. The performance of the nomogram was assessed using the receiver operating characteristic (ROC) curve and area under the curve (AUC) analyses.

**Results:**

The mean age of patients in the abdominal group was lower than that in the laparoscopic group (P < 0.05). Operation time and the number of dissected lymph nodes were higher in the laparoscopic group, whereas postoperative hospital stay and blood loss were lower (*P* < 0.05). The incidence of urinary retention in the laparoscopic group (53.7%) was significantly higher than in the abdominal group (38.2%; *P* < 0.05). Multivariate logistic regression results identified surgical approach, operation time, lympho-vascular invasion, and age as independent risk factors for postoperative urinary retention (*P* < 0.05). Calibration curve for the urinary retention nomogram aligned with the ideal curve. AUCs of the training and the validation sets were 0.819 (95% CI, 0.798-0.895) and 0.795 (95% CI, 0.782-0.824), respectively.

**Conclusion:**

Surgical approach, operative time, lymphovascular invasion, and age were identified as independent risk factors for urinary retention after hysterectomy in CC patients. The developed nomogram based on these independent risk factors had good accuracy and predictive ability.

## INTRODUCTION

Cervical cancer (CC) is a common malignant tumor in women.^1^ Usual treatment involves abdominal or laparoscopic radical hysterectomy.^2^ However, the incidence of postoperative complications is relatively high due to the extent of the surgical operation and inherent trauma of the procedure, especially in cases that necessitate pelvic lymph node dissections, which often lead to damage to the autonomic nerves that control bladder function and to urine retention.^3^,^4^ Therefore, long-term catheterization is often required after radical hysterectomy for CC.^5^,^6^ This study aimed to assess the occurrence of postoperative complications in CC patients who underwent abdominal and laparoscopic radical hysterectomies. The primary goal of the analysis was to identify risk factors of postoperative urinary retention in postoperative CC patients and to develop a nomogram model based on the identified factors. Such a model will provide a convenient and straightforward assessment method for clinicians and improve the quality of life of CC patients.

## METHODOLOGY

Clinical and pathological data of 438 patients with CC treated by the same team of physicians in Fujian Maternity and Child Health Hospital, China between December 2011 to December 2018 were retrospectively analyzed. Data were prospectively recorded during treatment and later analyzed for research purposes. Based on the method of radical surgery, patient records were divided into a conventional abdominal surgery group (196 cases) and a laparoscopic surgery group (242 cases). The sample size was determined using PASS 2025 (Release 25.0.2; NCSS, LLC, Kaysville, UT, USA) as follows: since the reported incidence of complications after radical cervical cancer surgery varies widely^15^,^16^, an estimated incidence of 50% with a 5% absolute precision and a 95% confidence level required a sample of 226 patients.

### Ethical approval:

The Ethics Committee of Fujian Maternity and Child Health Hospital reviewed and approved all studies involving human participants. Approval number 2024KY142; dated May 28, 2025.

### Inclusion criteria:

- Clear preoperative diagnosis of cervical malignant tumor.
- Clinical stage within the interval going from Ia2 to IIb, according to the FIGO 2018 staging standards.
- Preoperative chest X-ray, abdominal ultrasound, and other examinations show absence of distant metastases in the lungs, liver, abdominal cavity, or other organs.
- Extensive total hysterectomy ± bilateral adnexa + pelvic (and/or abdominal) lymph node dissection, with pathologically confirmed negative resection margins.
- Absence of grossly visible positive lymph node residues.

### Exclusion criteria:

- FIGO stage III and IV tumors.
- Intraoperative tumor peritoneal dissemination or presence of distant metastasis.
- Incomplete pathological diagnosis data.

### Surgical Methods:

All patients underwent radical hysterectomy combined with pelvic lymph node dissection. The same group of senior doctors, with more than 10 years of experience in CC operations, treated all patients, ensuring uniformity in surgical approaches.

### Variables and definitions:

The following data were collected for all the patients:

- Two gynecologic oncologists with senior professional titles determined the FIGO clinical staging based on pelvic examination findings.
- General clinical and pathology data, such as age, body mass index (BMI), history of previous operations, comorbidities, FIGO staging, neoadjuvant therapy, histology type, evidence of lymph node metastasis, parametrial invasion, and lympho-vascular invasion.
- Surgical indicators, such as surgical approach, operation time, intraoperative blood loss, and perioperative transfusions.
- *Postoperative recovery:* occurrence of urinary retention, other postoperative complications, and postoperative hospital stay length. The Clavien-Dindo grading standard^7^ was used to grade the severity of intraoperative and perioperative complications.

There are no standardized diagnostic criteria for postoperative urinary retention. In this study, postoperative urinary retention was defined as a patient being unable to urinate on their own or presenting residual urine of ≥100 mL more than 15 days after the surgical procedure, as described by Zuo et al.^8^ After removing the catheter, patients were instructed to empty their bladder as much as possible, and the residual urine volume was detected by B-ultrasound. If the residual urine volume exceeded 100 mL or the patient was unable to urinate autonomously, the catheter was replaced.

### Statistical analysis:

All statistical analyses were done using the Statistical Package for Social Science (SPSS) version 26.0 for Windows (IBM, Chicago, IL, USA). The results are expressed as percentages or as means ± standard deviation (SD), using *t*-tests. Count data are expressed as rates and analyzed using χ^2^ tests. Risk factors for urinary retention were assessed using univariate and multivariate analyses with logistic regression. The R 4.3.2 software, combined with the rms package, was used to analyze data and develop a postoperative urinary retention risk prediction nomogram. Patients were randomly assigned to a training (70%, 306 cases) or a validation set (30%, 132 cases). External validation was performed using the validation set; receiver operating characteristic (ROC) and calibration curves were used to evaluate the predictive value of the nomogram for postoperative urinary retention. *P* values < 0.05 were statistically significant.

## RESULTS

The average age of patients in the abdominal group (46.04±7.80 years) was significantly lower (*P* = 0.037) than in the laparoscopic group (47.71±8.73 years). There were no statistically significant differences between the two groups in terms of BMI, history of previous surgery, preoperative comorbidities, FIGO staging, preoperative treatment, neoadjuvant blood transfusion, histology type, or presence of lympho-vascular invasion or parametrial invasion (Table-I).

**Table-I:** Baseline clinical and pathological characteristics of patients in the two groups

	Abdominal surgery (n=196)	Laparoscopic surgery (n=242)	P value
Age (years)	46.04±7.80	47.71±8.73	0.037*
BMI (kg/m^2^)	23.04±2.92	22.69±3.14	0.234
Comorbidities, n (%)			0.299
Yes	72 (36.7)	113 (46.7)	
No	124 (63.3)	129 (53.3)	
Previous abdominal surgery, n (%)			0.776
Yes	33 (16.8)	35 (14.5)	
No	163 (83.2)	207 (85.5)	
Perioperative transfusion, n (%)			0.310
Yes	6 (3.1)	3 (1.2)	
No	190 (96.9)	239 (98.8)	
Histology subtype, n (%)			0.754
Adenocarcinoma	22 (11.2)	34 (14.0)	
Squamous-cell carcinoma	161 (82.1)	196 (81.0)	
Adenosquamous carcinoma	11 (5.6)	10 (4.1)	
Others	2 (1.0)	2 (0.8)	
Stage of disease, n (%)			0.549
IA2	2 (1.0)	5 (2.1)	
IB	129 (65.8)	166 (68.6)	
IIA1	31 (15.8)	27 (11.2)	
IIA2	25 (12.8)	36 (14.9)	
IIB	9 (4.6)	8 (3.3)	
Lymphovascular invasion, n (%)			0.719
Yes	68 (34.7)	80 (33.1)	
No	128 (65.3)	162 (66.9)	
Parametrial extension, n (%)			0.591
Yes	5 (2.6)	9 (3.7)	
No	191 (97.4)	233 (96.3)	
Neoadjuvant therapy, n (%)			0.080
No therapy	120 (61.2)	163 (67.4)	
Chemotherapy	52 (26.5)	64 (26.4)	
Radiotherapy	24 (12.2)	15 (6.2)	

BMI: body mass index,

* statistical significance (P<0.05)

The laparoscopic operations were associated with a longer mean operation time (*P* < *0.001)* and more lymph node dissections (*P* = 0.013) than the abdominal operations (Table-II). The mean postoperative hospital stay and intraoperative blood loss of the laparoscopic group were considerably shorter and lower (respectively) than those of the abdominal group (*P* < 0.001).

**Table-II:** Operative characteristics of patients in the abdominal and laparoscopic surgery groups.

	Abdominal surgery (n=196)	Laparoscopic surgery (n=242)	P value
Operation time (min)	257.43±40.79	293.31±55.49	<0.001*
Blood loss volume (mL)			<0.001*
<482	52 (26.5)	232 (95.9)	
≧482	144 (73.5)	10 (4.1)	
Day of first flatus (days)	2.45±0.74	1.88±0.50	<0.001*
Postoperative day (days)	11.97±4.00	10.60±3.53	<0.001*
Resected LNs (n)	27.04±9.75	29.66±11.90	0.013*
Positive LNs, n (%)			0.518
Yes	35 (17.9)	37 (15.3)	
No	161 (82.1)	205 (84.7)	

LN: lymph nodes;

* statistical significance (P<0.05)

The overall incidence of postoperative complications (excluding urinary retention) was similar in the two groups. The incidence of urinary retention in the laparoscopic group was 53.7%, significantly higher than that in the abdominal group (38.2%; *P* < 0.05). There were no significant differences between rates of other mild or severe complications in the two groups (*P* > 0.05). No mortalities within 30 days after surgery were reported in either group (Table-III). Moreover, the logistic regression analysis using indicators with statistically significant differences in the univariate comparison between the two groups identified surgical approach, operation time, lympho-vascular invasion, and age as independent risk factors affecting postoperative urinary retention after radical surgery for CC (*P* < 0.05, Table-IV).

**Table-III:** Postoperative morbidity and mortality in the abdominal and laparoscopic surgery groups

	Abdominal surgery (n = 196)	Laparoscopic surgery (n = 242)	P- value
Urinary retention	75 (38.2)	130 (56.2)	0.014*
General complications	39 (19.9)	48 (19.8)	0.467
Vaginal cuff dehiscence	0	1 (0.4)
Vesicovaginal fistula	0	1 (0.4)
Ureteral fistula	0	2 (0.8)
Inflammatory intestinal obstruction	2 (1.0)	0
Wound infection	0	1 (0.4)
Lymphatic fistula	1 (0.5)	1 (0.4)
Pelvic infection	11 (5.6)	9 (3.7)
Urinary tract infection	10 (5.1)	10 (4.1)
Venous thromboembolism	4 (2.0)	11 (4.5)
Lymphedema	7 (3.6)	6 (2.5)
Pulmonary infection	3 (1.5)	6 (2.5)
Intraperitoneal bleeding	1 (0.5)	0
Clavien-Dingo classification			0.537
II	35 (17.9)	39 (16.1)	
IIIA	2 (1.0)	5 (2.1)	
IIIB	2(1.0)	4(1.7)	
IV	0	0	
V	0	0	

* statistical significance (P<0.05).

**Table-IV:** Multivariate logistic regression analysis of risk factors for postoperative urinary retention.

	B	P value	OR	95% CI
Surgical approach	0.485	0.04	1.624	1.021-2.583
Operation time	0.471	0.001	1.601	1.203-2.131
Age	0.131	<0.001	1.141	1.105-1.177
Lympho-vascular invasion	1.149	<0.001	3.154	1.946-5.112

**Fig.1:**
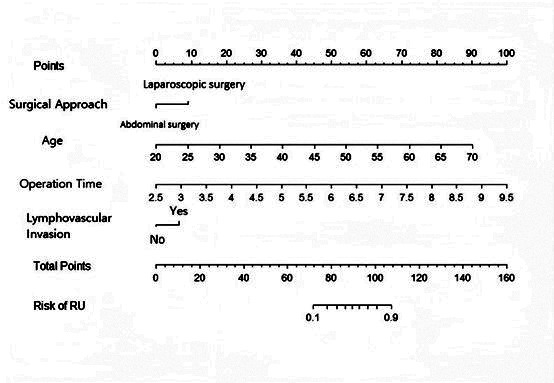
Nomogram for predicting the risk of postoperative urinary retention after radical surgery for cervical cancer. The nomogram includes four independent predictors: surgical approach, age, operation time, and lymphovascular invasion. Each variable corresponds to a specific point value (top “Points” scale). By summing the individual scores for each predictor. The total score (“Total Points” scale) is determined by summing the individual scores for each predictor. The probability of urinary retention (RU) is then determined, with higher total points indicating an increased predicted risk of postoperative urinary retention.

**Fig.2:**
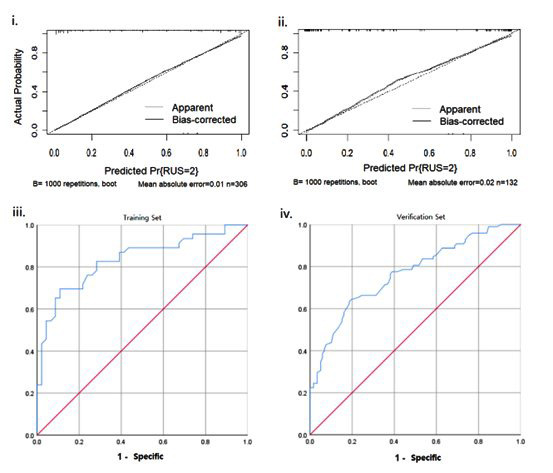
Calibration and discriminative performance of the nomogram model for predicting urinary retention. (i) Calibration curve of the training set (n = 306), showing the relationship between the predicted and actual probabilities of urinary retention (UR). The solid line represents the apparent performance, while the dashed line indicates the bias-corrected performance obtained by bootstrapping with 1000 repetitions (mean absolute error = 0.01). (ii) Calibration curve of the verification set (n = 132), showing good agreement between predicted and observed outcomes after bias correction (mean absolute error = 0.02). (iii) Receiver operating characteristic (ROC) curve of the training set, demonstrating the model’s discriminative ability to distinguish between patients with and without urinary retention. (iv) ROC curve of the verification set, confirming the predictive performance of the model in the external dataset. The diagonal red line represents the reference line for random prediction.

A nomogram model for predicting the occurrence of postoperative urinary retention was then established based on selected independent risk factors (Fig.1). The model was internally validated, and external validation was completed using a validation set. The calibration curves of both sets were aligned with the ideal curve. The predicted values are consistent with the actual measurements (Fig.2). The area under the ROC curves for the training and validation sets was 0.819 (95% CI, 0.798-0.895) and 0.795 (95% CI, 0.782-0.824), respectively (Fig.2).

## DISCUSSION

The study demonstrated a high incidence of postoperative urinary retention after radical surgery for CC. The laparoscopic approach was associated with a considerably higher risk of postoperative complications than the abdominal approach. The study identified independent risk factors for urinary retention, including surgical approach, operative time, and the development of lymphovascular invasion. The developed nomogram demonstrated good predictive value, making it a potentially useful tool for providing timely, accurate treatment.

The conclusions of the laparoscopic approach to CC (LACC) study have led to the adoption of open surgery as the standard of care for early CC treatment, limiting the use of laparoscopic approaches in CC.^9^,^10^ However, laparoscopic operations are widely used in many clinical settings and accepted by both doctors and patients for the treatment of CC. An increasing number of studies have demonstrated that, for certain CC stages, the oncological outcomes of laparoscopic surgery are equivalent to those of open surgery.^11^-^13^ Therefore, while there is controversy surrounding minimally invasive surgery for CC, continuing to explore the application of laparoscopic operations in its treatment is crucial to standardize and improve surgical procedures, reduce intraoperative and postoperative complications, and enhance oncological outcomes for patients.

The laparoscopic approach allows surgeons to clearly visualize the anatomical layers. Furthermore, magnification of the visual field during the procedure accurately displays the delicate vascular, nerve, and fascial structures involved, potentially improving the accuracy of the operation.^14^ This study shows that laparoscopic surgery can significantly reduce the intraoperative blood loss, more thoroughly clear lymph nodes, and shorten postoperative hospital stay lengths. The longer operation times with the laparoscopic approach may be due to the complexity and high precision of the procedure. The analysis of postoperative complications according to the Clavien-Dindo classification in this study showed that the overall incidence of postoperative complications for patients with CC undergoing laparoscopic operations was comparable to that of the patients undergoing abdominal operations (19.8% *vs* 19.9%, *P* = 0.467), further confirming that this approach is safe and feasible.

The extent of the surgical procedure necessary for the CC resection and the resulting inherent tissue trauma, regardless of whether the operation is laparoscopic or abdominal, leads to a high incidence of postoperative complications. Bladder and rectal dysfunctions are common; they influence the rate of postoperative recovery and reduce the quality of life of patients. The reported incidence rates of complications after radical CC operations range between 4.39% and 70%.^15^,^16^ In this study, among 438 patients who underwent radical CC resections, 206 developed bladder dysfunction, for an incidence of 47.0%, consistent with previous data. These results emphasize the need to reduce postoperative urinary retention while improving the surgical curative rate. Urinary retention and its impact on the patient’s quality of life has become a focus of research.^17^,^18^ Studies have shown that early postoperative urinary retention after radical surgery for CC is significantly associated with the surgical operation, as the nerves controlling the bladder may inevitably be injured during surgery.^19^-^21^

In this study, the independent risk factors for postoperative urinary retention included age, surgical approach, operation time, and lymphovascular invasion, all of which are directly affected by the surgical procedure. Patients with lymphovascular invasion are often in advanced disease stages and present a higher frequency of lymph node metastasis.^22^ During radical surgeries, the range of lymph node dissection is extensive, and the probability of intraoperative injury to the nerves controlling the bladder is high.^23^ The longer operation times of laparoscopic operations may increase the traction and stimulation of the bladder during the procedure, and common injuries such as compressions and contusions can exacerbate bladder paralysis or bladder muscle injuries caused by surgical instruments. Therefore, efforts to shorten operating times may help reduce intraoperative bladder traction and stimulation. In addition, opting for radical surgery that protects the pelvic autonomic nerves may be preferable for suitable cases^24^ to reduce intraoperative nerve damage and improve the postoperative urinary function. Multiple studies have confirmed that age may increase the risk of postoperative urinary retention.^25^,^26^ This may be due to the atrophy or fibrosis of the bladder muscles that occur with age, leading to decreased contractility of the detrusor muscle. Additionally, older patients may have a weakened immune system and require prolonged bed rest, both of which may increase the risk of urinary tract infections. Moreover, indwelling catheters are associated with an increased risk of urinary tract infections, which can aggravate urinary retention.^27^ At the same time, urinary retention can be the cause of urinary system infections. Therefore, these factors form a vicious cycle that may lead to severe bladder dysfunction.^28^,^29^

The study used multivariate regression to develop a nomogram that demonstrated good predictive value. This model has significant clinical application, as it may allow clinicians to sum up the scores of each independent risk factor for a comprehensive assessment of the risk of postoperative urinary retention, to evaluate the duration of postoperative catheter retention, to reduce the patient discomfort caused by repeated insertion and removal of the catheter, and to reduce the incidence of urinary tract infections.

### Limitations:

This was a retrospective single center study with an inherent selection bias risk. However, strictly formulated inclusion and exclusion criteria and a sufficient number of cases collected ensured that the experimental and control groups truly reflect the basic situation of the disease in the population. Surgeon experience, perioperative bladder management protocols and the absence of patient-reported outcomes should also be fully considered, as they may affect the quality of surgery and the occurrence of complications. The internal validation on the data was performed using patient cohort from a single center. Future studies should obtain multi-center data for external validation of the model.

## CONCLUSION

The incidence of postoperative urinary retention after radical surgery for CC is high, especially after laparoscopic surgery. This study identified age, surgical approach, operation time, and lymphovascular invasion as independent risk factors for urinary retention, and the nomogram we generated demonstrated good predictive value. This prediction model may be clinically useful in providing timely, accurate treatment for patients with CC undergoing surgery.

### Authors’ contributions:

**TC:** Literature search, study design, and manuscript writing.

**LZ, YS, and HY:** Data collection, data analysis and interpretation, Critical review.

**TC:** Critical analysis, Manuscript revision, validation and is responsible for the integrity of the study.

All authors have read and approved the final manuscript.
